# A self-healing ferroelectric liquid crystal electro-optic shutter based on vertical surface-relief grating alignment

**DOI:** 10.1038/s41467-021-24953-5

**Published:** 2021-08-05

**Authors:** Peter J. M. Wyatt, James Bailey, Mamatha Nagaraj, J. Cliff Jones

**Affiliations:** 1 School of Physics and Astronomy, University of Leeds, Leeds, UK; 2 Dynamic Vision Systems Ltd., Leeds Innovation Centre, Leeds, UK

**Keywords:** Electrical and electronic engineering, Electronic devices, Displays, Liquid crystals

## Abstract

Ferroelectric liquid crystals remain of interest for display and spatial light modulators because they exhibit significantly faster optical response times than nematics. However, smectic layers are sensitive to shock-induced flow and are usually permanently displaced once a well-aligned sample is disrupted, rendering such devices inoperable. We introduce a vertical alignment geometry combined with a surface-relief grating to control both the smectic layer and director orientations. This mode undergoes “self-healing” of the smectic layers after disruption by shock-induced flow. Sub-millisecond switching between optically distinct states is demonstrated using in-plane electric fields. Self-healing occurs within a second after being disrupted by shock, wherein both the layer and director realign without additional external stimulus. The route to material improvements for optimised devices is discussed, promising faster spatial light modulators for high-speed adaptive optics, micro-displays for virtual/augmented reality and telecommunications with inherent shock stability.

## Abstract (web summary)

Ferroelectric liquid crystals (FLCs) have faster optical response times than nematic crystals, but they are also less robust to external shock. Here the authors develop an FLC geometry that reduces the sensitivity to external disruption through self-healing, making them more stable for applications.

## Introduction

Ferroelectric liquid crystals (FLCs) were first studied as candidates for television and computer monitor displays in the late 1980s and 1990s due to their sub-microsecond switching times and inherent bistability^1–4^. The surface stabilised FLC (SSFLC)^1^ seemed suited as an alternative to nematic-based LCDs since high complexity passive displays could be made without thin-film-transistors (TFTs) at each pixel^5^. Although bistable, SSFLC displays achieved 256 grey-levels using two spatial dither bits combined with four bits of temporal dither at 60 Hz, allowed by the FLCs sub-millisecond optical response times^4^. Even following large area TFT panel commercialisation, interest remained in the fast response times offered, with so-called “V-shaped” switching panels using a combination of TFT and a monostable FLC with an analogue electro-optic response^5,6^. However, FLCs in such devices are susceptible to shock, whereby induced viscous flow disrupts the well-aligned smectic layers causing optically visible damage that is effectively permanent^7,8^. This weakness made FLC unsuited for large area displays. However, such fast speeds still remain highly desirable today, for instance enabling frame sequential colour optics in projector display applications. Liquid-Crystal-on-Silicon (LCoS) spatial light modulators based on FLC are commercially successful^9–12^, and although are less sensitive to shock, shock-insensitive modes remain important to explore and develop for the next generation of portable and wearable devices. They must exhibit ultra-high resolution, be light weight with a low power consumption, whilst demonstrating high diffraction or transmission efficiencies^13^. In addition, novel geometries must have a relatively low-fabrication complexity to be commercially suitable for upscaling by using methods such as photoalignment^14^ or nano-imprint lithography^15^.

Shock-stability in FLCs has been improved using methods including polymer stabilised FLCs (PSFLCs)^14,16^, and utilising high pitch FLCs in a natural helical alignment that is not defined by the surface-alignment, demonstrated by the deformed helix (DHFLC)^17^ and electrically suppressed helix (ESFLC) modes^18,19^. In addition, homeotropic^20^, or vertical alignment modes of FLCs has been explored to resist shock induced effects, such as the vertically aligned FLC (VAFLC)^21^ and the vertically aligned deformed helix FLC (VA-DHFLC)^22^, of which both utilise interdigitated electrodes (IDEs). These VA modes prove easier to obtain uniform alignment than the SSFLC, while exhibiting much higher contrast ratios. These approaches either rely on the geometry providing some resistance to shock or being constantly electrically addressed to appear bright or dark, albeit with reduced contrast. In this work, a method that allows the material to self-heal after becoming disrupted is presented.

Profiled surfaces and surface-relief gratings have been established as alignment layers since the 1970s^23^, where alignment is induced to the nematic director, **n**, as originally proposed by Berreman^24^. Such surfaces are used in this work to impart a preferred alignment direction to the smectic layer normal, **a**, and the **c**-director of FLCs which defines the in-layer-plane orientation of the FLC **n**-director. More complex geometries of surface-relief gratings for nematic liquid crystals have seen commercial success with the zenithal bistable display (ZBD)^25^, where deep homeotropic surface-relief gratings are implemented to induce surface bistability in a TN display^26,27^, whilst only introducing one additional fabrication step, namely, introducing the surface-relief gratings to the manufacturing process^28^.

In this work, FLCs are aligned using weakly anchored homeotropic surface-relief gratings and shown to produce a device geometry that is resistant to a mechanical shock through self-healing with no further required stimulus. This combines the durability of nematic liquid crystals whilst realising the sub-millisecond response times that are required for next generation displays and photonic devices. The geometry is termed vertical-grating aligned ferroelectric liquid crystal (VGA-FLC). The results prove promising for implementation in electro-optic shutters and LCoS display technologies, whilst requiring little modification to established industrial processes for display fabrication^29,30^.

## Results

### Device theory and operation

**Fig. 1:**
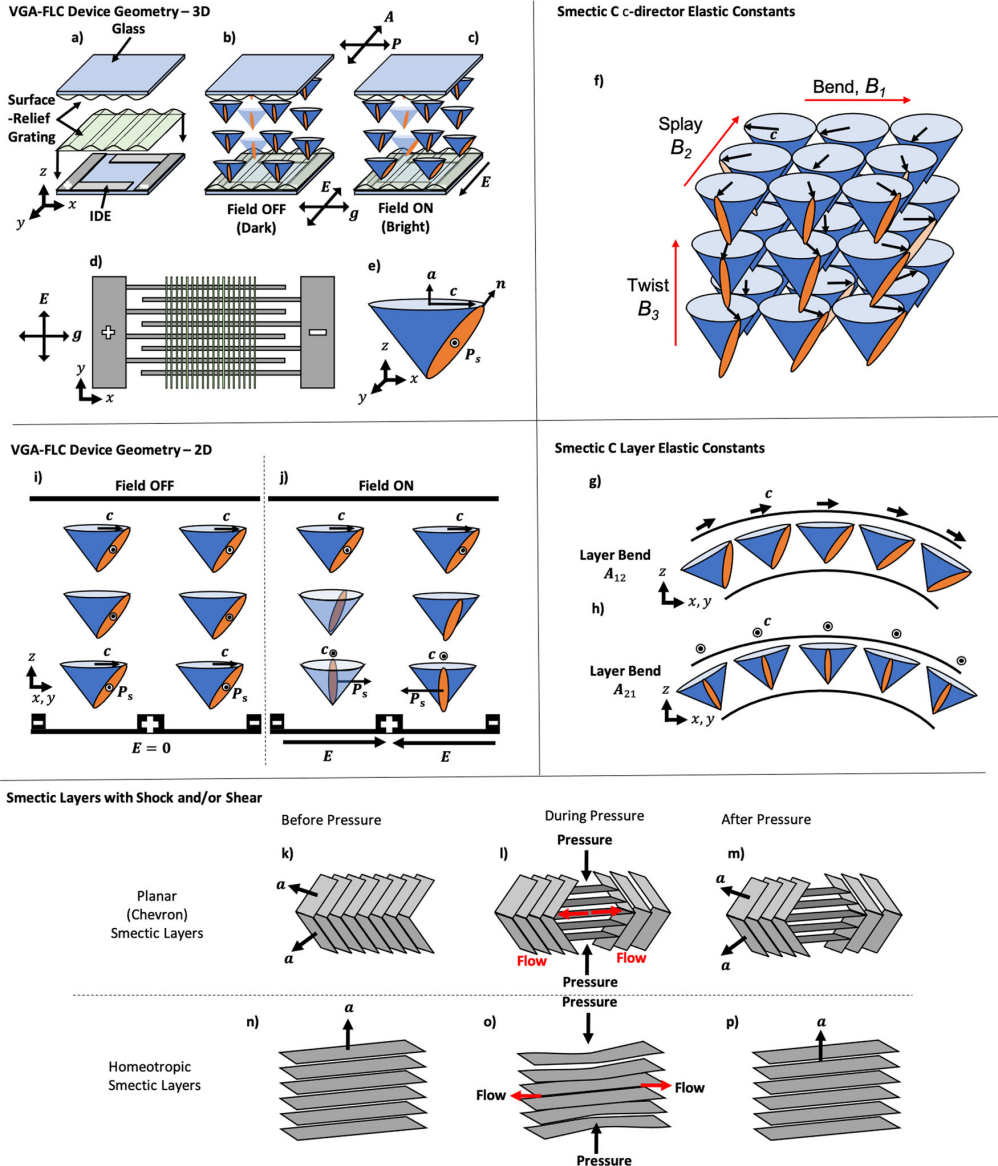
The geometry of the vertical grating-aligned ferroelectric liquid crystal device (VGA-FLC). **a**–**e** Schematic diagrams and operating principles of the VGA-FLC device geometry. **a** 3D schematic of the surface relief grating and its arrangement with respect to the interdigitated electrode (IDE). **b** Device with no applied electric field (OFF/dark state), where the **c**-director is aligned homogeneously through the cell. **c** Schematic of the effect of applying an electric field across the IDE to rotate the **c**-director 90° at the lower surface and therefore a twisted configuration through the cell. On removal of this field, the device relaxes to the homogeneous **c**-director profile of (**b**). **d** Plan view of the grating orientation with respect to the IDE. **e** Definitions for the FLC **c**-director, **n**, and the spontaneous polarisation, **P**_s_. **f**–**h** Definitions of the **c-**director elastic constants. **f** Shows **c**-director bend (B_1_), splay (B_2_), and twist (B_3_) for uniform layers. **g** and **h** Show the possible orientations of the **c**-director with respect to a sinusoidal undulation of the smectic layer normal **a** in a SmC phase. In either case, the solution shown is accompanied by a second possibility with the **c** director at 180°. Shown are one of the two solutions for (**g**) **c⊥g**, when A_12_ « A_21_; and (**h**) **c || g**, when A_12_ » A_21_**. i** and **j** A 2D schematic of the field on either side of an IDE where (**i**) has no applied field, and (**j**) has an applied field twisting the **c**-director by 90° through the cell in opposite direction across the electrode. **k**–**p** The response of both planar and homeotropic oriented smectic layers when exposed to a mechanical shock, where **a** is the layer normal. When initially in **a** planar orientation (**k**), pressure disrupts the orientation of the layers to a homeotropic alignment geometry in the affected region (**l**), which remains after force removal (**m**). In the homeotropically aligned state (**n**) the layers compress, resulting in compression and therefore in-plane flow of the FLC (**o**), withstanding significant disruption to the alignment of the smectic layers (**p**).

A simple geometry for FLC electro-optic shutters is presented in Fig. 1a–e, based on sub-micron amplitude and micron-scale pitch surface-relief gratings. The surface-relief gratings are embossed^28,31^ into a photopolymer on top ITO in-plane switching (IPS) electrodes on one substrate, and on to plane glass on the opposing substrate, given the appropriate spacing using plastic beads in the conventional fashion, and capillary filled with the FLC material in its nematic phase. The device is designed to controllably align the FLC in a surface-stabilised vertical alignment geometry using homeotropic surface treatment, whereby the layers are uniformly parallel to the substrate plane (Fig. 1f), except close to the grating surface where there are microscopic oscillations that induce the desired **c**-director alignment, shown in Fig. 1g, h. This is essential to achieve good optical contrast whilst enabling a geometry that is insensitive to shock-induced flow. The surface-relief gratings are surfactant treated to induce a homeotropic, or vertical, alignment^32^ to the FLC layer normal, **a**, where the layer and substrate normal are parallel to one another. The homeotropic anchoring energy of the surfactant was controlled through the exposure time to the surfactant vapour and was estimated to be (1.0 ± 0.2)×10^−4^ J/m^2^ by comparison with the previous calibration done by Jones et al. ^32^. Fig. 1i, j provides a schematic indicating how the director alignment and electric field are used to switch the device between a dark and a bright state. The ON state must be electrically addressed to switch, while the OFF state can either be electrically addressed or allowed to relax back naturally. This alignment promises greater shock stability than observed in planar, non-helical geometries as seen in similar devices^21,22,33^, enabled by the initial alignment of the smectic layers relative to the direction of the induced liquid flow. A schematic diagram in Fig. 1k–p describes how the smectic layers are affected by shock-induced flow in a planar and homeotropic alignment. On mechanical shock, any induced flow remains in the cell plane such that there is little or no distortion to the average orientation of **a**, and mainly governed by reorientation of the **c**-director within the layer in the direction of flow^34–37^. The **c**-director is found to naturally reorient back to its initial alignment to once again align with **g**, being the lowest energy state of the system. This is observed as a self-healing mechanism of the optical texture as opposed to a shock-resistance whereby the containment prevents severe layer reorientation. The surface-relief gratings are low-amplitude (<1 µm) and low-pitch (4 µm) to minimise distortions to the static smectic layers yet remaining effective at restoring the desired **c**-director alignment following a mechanical shock or electrical address.

**Fig. 2:**
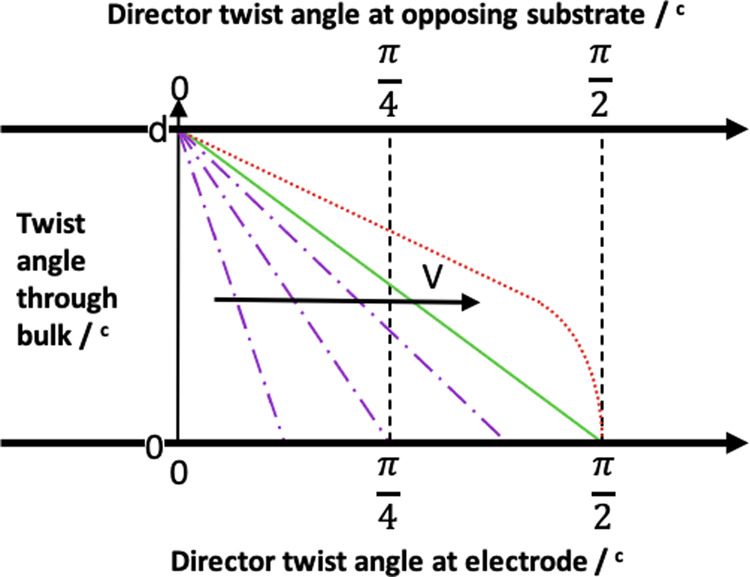
Director twist with applied voltage. A schematic diagram showing how the total twist of the **c**-director is expected to vary through the cell (*z*-direction) as a function of applied voltage. The interdigitated electrodes are located on the bottom axis of the diagram. The purple dotted and dashed lines show a twist less than 90°, or $\frac{\pi }{2}$ radians. The green solid line shows an optimum 90° twist through the device. The red dotted line shows an over-twist of the **c**-director, which results in a reduced transmission.

In the OFF state without an applied electric field, the **c**-director of the FLC is aligned by the surface-relief gratings on opposing surfaces and lies perpendicular to **g**, shown Fig. 1b, i. This results in no transmitted light when oriented between crossed polarisers, with **g** parallel to one polariser. The desired optical contrast requires that the natural helicity of the FLC is unwound in the OFF state by the opposing grating surfaces, to suppress the natural uniform twist of the **c**-director through the cell. This is satisfied when the FLC helical pitch is^38^

1 $$P \, > \, 4d,$$

where *d* is the cell gap, and a sufficiently large in-plane anchoring strength of **c** from the grating alignment is assumed. The ON state is obtained through the application of an electric voltage to the IDEs, creating an in-plane electric field over the FLC. The FLC **c**-director close to the IDE substrate begins to rotate in-plane, aligning the **P**_s_ vector parallel to the field direction. With increasing electric field, further **c**-director rotation is observed through the cell. When it rotates through an angle of 90° through the cell, the bright state of the device is obtained, or ON state, shown schematically in Fig. 1c, j. On removal of this electric field the **c**-director relaxes back to once again align with the grating, restoring the OFF, or dark, state. This effect occurs within the smectic layers as the **P**_s_ and dielectric biaxiality are both in the plane of the field. Therefore, **n** reorients within the smectic layers without disruption as the torque remains in the plane of the layers. A carefully selected restoring pulse of opposite sign to the ON pulse can be applied to quickly switch the device back to its initial OFF state. For simplicity we consider the **c**-director of the device only, wherein the IDEs induce a transition from a uniformly oriented **c**-director from one surface to the other in the OFF state, and the **c**-director reorients towards 90° close to the IDE surface when a field is applied. The **c**-director is approximated to a twisted-nematic type of configuration in these circumstances. For some intermediate voltage, the twist of the **c**-director is 90° so that the device’s bright state between crossed polarisers satisfies the Gooch–Tarry equation for the first minimum^39,40^:

2 $$\triangle {n}_{{{eff}}}d=\frac{\sqrt{3}}{2}\lambda ,$$

where *d* is the cell gap, Δ*n*_*eff*_ is the effective birefringence of the LC, and *λ* is the wavelength of incident light. Figure 2 represents how the total twist through the device changes with an applied electric field by varying the voltage, assuming a linear change in the magnitude of the field through the bulk of the FLC layer. The change in voltage is analogous to how the twist changes with time. Ultimately, this relatively simple geometry has led to a working prototype that demonstrates both resistance to mechanical shock as well as sub-millisecond optical response times.

### Alignment of the FLC in the VGA-FLC geometry

**Fig. 3:**
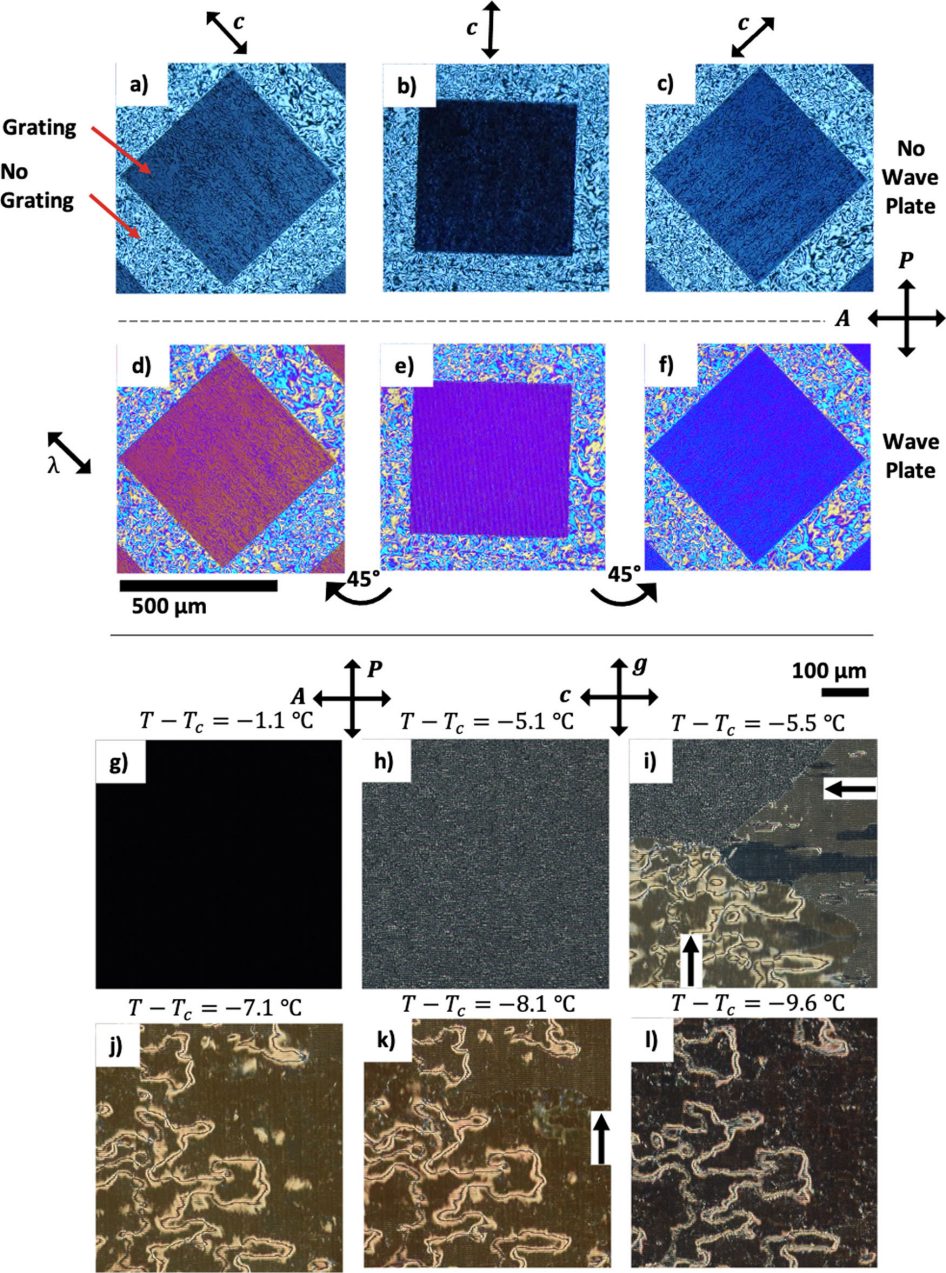
Polarising optical microscope (POM) images of the SmC(*) alignment in the VGA-FLC device. **a**–**f** The alignment effects of homeotropic surface-relief gratings on the smectic **c**-director on a single grating with pitch of 4 µm and peak-to-peak amplitude of 0.3 ± 0.1 µm SU-8 grating, with *d* = 6.4 ± 0.6 μm, and filled with SCE13-R. **a**–**c** Rotations of the gratings between crossed polarisers, showing regions with no gratings showing a schlieren texture, and regions with gratings inducing a preferred **c**-director orientation. **d**–**f** A full wave plate oriented at 45° to crossed polarisers reveals the **c**-director orientation is perpendicular relative to the grating vector **g**. This reveals that the smectic layers form an A_21_ elastic deformation. **g**–**l** POM images on cooling VGA-FLC Device^1^ (Table 1) at 0.5 °C min^−1^ into the SmC(*) phase. **g** *T* = 59.7 °C (Δ*T* = 1.1 °C) The dark texture at the SmA to SmC* transition before undulating textures appear, showing good homeotropic alignment. **h** *T* = 55.7 °C (Δ*T* = 5.1 °C) Carpet-like textures arise below the SmA to SmC(*) transition. **i** *T* = 55.2 °C (Δ*T* = 5.6 °C) Carpet textures dissipate behind a dislocation line of layer formation. The number of defects on the grating texture depends on the direction of layer formation flow relative to the grating vector **g**. **j** *T* = 53.7 °C (Δ*T* = 7.1 °C) Texture free of undulations. **k** *T* = 53.7 °C (Δ*T* = 7.1 °C) Undulations continuously appear and dissipate. **l** *T* = 51.2 °C (Δ*T* = 9.6 °C) The texture on further cooling become optically dark and well aligned, away from the disclinations.

The **c**-director alignment of the devices was examined using polarising optical microscopy (POM), shown in Fig. 3a–c. The FLC SCE13-R was used in these experiments. A racemic mixture was chosen to ensure no effects arising from helicity were observed. The optical textures of both the nematic and SmA phases remained uniformly dark on cooling, including on rotation between crossed polarisers, indicating strong homeotropic alignment of the liquid crystal director. In addition, this shows that the SmA layers are not disrupted by local compressions caused by the surface relief grating and that any distortion or resulting defects are limited to the surface layer. This is because the homeotropic anchoring energy of the surfactant is relatively weak, allowing the layers to align with minimal distortion around the surface relief structure. With the grating positioned parallel to the polariser for a dark state, the texture becomes marginally brighter during further cooling into the SmC phase. This texture becomes brighter on rotation of the sample between crossed polarisers, and brightest at 45° between crossed polarisers. This shows that the **c**-director is controllably orientated by the surface-relief grating, whilst maintaining a homeotropic alignment of the smectic layers. This is analogous to how a nematic LC would align on a planar grating or rubbed polymer, where the director, **n**, lies perpendicular to **g**, or parallel to a rubbing direction^24^. In comparison, a Schlieren texture is obtained for areas without surface-relief gratings as there is no preferred **c**-director orientation, just the homeotropic condition on **a**.

The in-plane anchoring strength *w*_*β*_ of a nematic LC on a planar-aligned sinusoidal surface-relief grating is given by the Berreman equation^24^:

3 $${w}_{\beta }=\frac{1}{4}\sqrt{{K}_{11}{K}_{33}}{\alpha }^{2}{\left(\frac{2\pi }{L}\right)}^{3},$$

where *K*_11_ and *K*_33_ are the splay and bend elastic constants of a nematic liquid crystal, *α* is the peak-to-peak amplitude, and *L* is the pitch of the grating. In the case of a SmC LC (either SmC or SmC*), a one constant approximation can be used^41^, but is otherwise assumed to be analogous to the nematic case. For the gratings studied in this work, the in-plane anchoring strength of the surface-relief grating is in the order of ${10}^{-6}{\mathrm {N}}{{\mathrm {m}}}^{-1}$, which is considered weak anchoring, but is shown to be sufficient to controllably align **n**, and subsequently ***c***, perpendicular to the grating vector, **g**. The effect of varying the anchoring strength of the surface-relief grating by varying grating amplitude and pitch has not been studied. However, a reduced pitch is expected to increase the uniformity of the alignment through an increase of the anchoring strength. Meanwhile, increasing the grating amplitude is expected to result in increased layer undulations in the static state, causing defect formation and loss of optical contrast.

The VGA-FLC geometry relies on the effect of the surface relief grating on both the 1D solid-like smectic layers and the 2D nematic-like **c**-director. We shall consider the case where the smectic layers form on cooling from a higher temperature nematic phase into the untilted SmA. The nematic ***n*** director field will deform to follow the topography of the grating close to the surfaces. The amplitude of this oscillation will decrease because *K*_33_ diverges as the SmA phase is approached. However, the layers will remain slightly compressible, allowing an elastic deformation. The microscopic observations show that the **c**-director lies perpendicular to that of the layer oscillation induced by the grating (Fig. 3). The energy density for a SmC system with compressible layers is rather complex. For simplicity the free energy, *w*, for an achiral SmC with incompressible layers is considered, as identified by Leslie, Stewart, Carlsson and Nakagawa^42^, with a particularly convenient form later given by^43^

4 $$w=	 \frac{1}{2}{A}_{12}({{{{{\boldsymbol{b}}}}}}\cdot \nabla \times {{{{{\boldsymbol{c}}}}}})^{2}+\frac{1}{2}{A}_{21}({{{{{\boldsymbol{c}}}}}}\cdot \nabla \times {{{{{\boldsymbol{b}}}}}})^{2}\\ 	+{A}_{11}\left({{{{{\boldsymbol{b}}}}}}\cdot \nabla \times {{{{{\boldsymbol{c}}}}}}\right)\left({{{{{\boldsymbol{c}}}}}}\cdot \nabla \times {{{{{\boldsymbol{b}}}}}}\right)+\frac{1}{2}{B}_{1}(\nabla \cdot {{{{{\boldsymbol{b}}}}}})^{2}\\ 	+\frac{1}{2}{B}_{2}(\nabla \cdot {{{{{\boldsymbol{c}}}}}})^{2}+\frac{1}{2}{B}_{3}\left[\frac{1}{2}\left({{{{{\boldsymbol{b}}}}}}\cdot \nabla \times {{{{{\boldsymbol{b}}}}}}\,+{{{{{\boldsymbol{c}}}}}}\cdot \nabla \times {{{{{\boldsymbol{c}}}}}}\right)\right]^{2}\\ 	+{B}_{13}\left(\nabla \cdot {{{{{\boldsymbol{b}}}}}}\right)\left[\frac{1}{2}\left({{{{{\boldsymbol{b}}}}}}\cdot \nabla \times {{{{{\boldsymbol{b}}}}}}\,+{{{{{\boldsymbol{c}}}}}}\cdot \nabla \times {{{{{\boldsymbol{c}}}}}}\right)\right]\\ 	+{C}_{1}\left(\nabla \cdot {{{{{\boldsymbol{c}}}}}}\right)\left({{{{{\boldsymbol{b}}}}}}\cdot \nabla \times {{{{{\boldsymbol{c}}}}}}\right)+{C}_{2}\left(\nabla \cdot {{{{{\boldsymbol{c}}}}}}\right)\left({{{{{\boldsymbol{c}}}}}}\cdot \nabla \times {{{{{\boldsymbol{b}}}}}}\right),$$

where **b** **=** **a** **×** **c**, and *A*_12_, *A*_21_, and *A*_11_ are elastic constants related to the bending of the smectic layers (see Fig. 1g, h), ${B}_{1},{B}_{2},{B}_{3}$ and *B*_13_ are the **c**-director bend, splay, twist and bend–twist elastic constants related to the orientation of the **c**-director within or across smectic layers (see Fig. 1f), and finally *C*_1_ and *C*_2_ are related to couplings of these aforementioned deformations. It should be noted that the vector **b** is parallel (or anti-parallel) to the direction of **P**_S_ if the material is chiral and hence ferroelectric. In terms of the elastic constants associated with the deformation of the smectic layers, the bending of the layers in this VGA-FLC geometry is an *A*_21_ eigen-deformation^43,44^, confirmed using POM and a full-wave plate as described by Fig. 3d–f. In other words, there is an undulating geometry induced into the smectic layers that follows the surface of the grating, and within the layers the **c**-director of the FLC lies perpendicular to the direction of this induced grating vector. For such an undulating layer profile over a 200 - 300 nm deep grating surface, defects must arise to lower the energy associated with such A_21_ eigen-deformations, as continuous undulations propagating through the bulk of the LC are energetically unlikely. Any inherent sinusoidal layer undulations induced into the static equilibrium state for a SmA (for simplicity) at its boundary can be described by the energy density term^45^:

5 $${w}_{A}=\frac{1}{2}{K}_{1}{\left(\frac{{\partial }^{2}u}{\partial {x}^{2}}\right)}^{2}+\frac{1}{2}\bar{B}{\left(\frac{\partial u}{\partial z}\right)}^{2}$$

where $u(x,y,z)$ is the vertical displacement of the layers relative to its initial position, $\bar{B}$ is the layer compression constant, and *K*_1_ is simply replaced for either *A*_12_ or *A*_21_ for the SmC(*) case. Introducing the boundary condition $u(x,0)={u}_{0}{{\sin }}({kx})$, defining the penetration length as^46^

6 $$l=\frac{1}{\Lambda {k}^{2}}$$

and the understanding that *z* decays to infinity gives the solution^46^

7 $$u\left(x,0\right)={u}_{0}{{\sin }}({kx}){{\exp }}\left(-\frac{z}{l}\right)$$

where $k=2\pi /{\lambda }_{c}$ is the wave number of undulation of period ${\lambda }_{c}$, with ${u}_{0}$ as a constant of layer undulation amplitude. For such a description to hold, $\left|{u}_{0}\right|\ll 1$ and must be smaller than the smectic layer thickness, or nonlinear effects require to be considered^45^. For the gratings considered in this work, the above limit is not held, as the penetration length of the undulations are *l* ~ 2 µm. So, we propose the formation of line defects that arise following the peaks and grooves of the grating, analogous to the smectic oily^47^ or soapy^48^ streak textures found in similarly undulating smectic textures. Such layer undulations over the surface-relief grating are thought not to extend far from the surface boundary due to the inherent requirement to lower the energy cost of such undulations, and so cannot be observed with POM. Away from the surface, the undulations decay to a length scale below the layer spacing and become continuous. It is the coupling of the **c**-director to the sinusoidal fluctuation of the layer normal, **a**, through the A_21_ Eigen-deformation aided by the surface-relief grating that causes alignment of the **c**-director. Because only two domains are found, with **c** either at +90° or −90° to **g**, the condition ${A}_{21} \, < \, {A}_{12}$ is met. This is the first time that such an alignment has been shown for a SmC or SmC* liquid crystal.

Figure 3g–l shows the alignment of the surface-stabilised SmC* phase on cooling from the SmA. The initial texture of the SmC* in the VGA-FLC geometry on cooling from the SmA may be described as carpet-like, with textures induced by and following the periodicity of the grating, as shown in Fig. 3h. On further cooling, a domain wall corresponding to texture reorientation sweeps across the sample, of which the direction follows the temperature gradient of the device, indicate with black arrows in Fig. 3i. This defect-led reorientation changes the texture from the initial carpet-like state into a more uniformly dark alignment state, similar to the planar rubbed nematic texture, but SmC(*). The direction of this temperature gradient relative to the grating vector **g** determines the number of defects that appear in the new texture, as the ***c***-director aligns with its direction.

It is known that the cone angle of the SmC* material increases with decreasing temperature, following the temperature dependence^49^:

8 $${\theta }_{{\mathrm {C}}}={\theta }_{0}{({T}_{{\mathrm {C}}}-T)}^{\gamma },$$

where the critical exponent *γ* is typically $0.35\le \gamma \le 0.5$, and the material-dependent constant *θ*_0_ is typically about 5°. As the sample is cooled through the SmC(*) phase, the component of the birefringence in the plane of polarisation for normally incident light increases both due to the inherent change in Δ*n* in response to increasing order parameter *S*, but also due to the increasing cone angle *θ*_C_ through Eq. (3). Due to the *A*_21_ layer deformations, there will also be a minor increase in transmission as the cone angle increases, which will again increase if increasing the magnitude of the undulating texture. This apparent first-order texture transition results from the discontinuous change in layer orientation from a non-equilibrium tilted layer configuration, to one where the layers are parallel to the plane of the device. As the cone angle increases on further cooling, so increases the number of smectic layers between the cell walls. Rather than all of the layers adjusting spontaneously to create a new layer to satisfy the induced homeotropic condition, it is easier to tilt the layers slightly to retain the same number of layers but with the higher cone angle. These undulations are stresses caused by thermal contractions resulting from the energy cost associated with creating new smectic layers to fill the new space resulting from the reducing layer shrinkage. After sufficiently high disruption to the layers, or sufficient time, the layers return from this metastable state to the uniform planar condition with the new cone angle. Such observations have been seen previously for homeotropic aligned FLCs with thermally induced double undulations, caused by stresses from thermal contractions of the smectic layers^50^. Compressions in the SmC(*) phase can be introduced as an additional term to the free energy approximation^45^:

9 $${w}_{{{comp}}}=\frac{1}{2}\bar{B}{\left(\frac{\partial u}{\partial z}\right)}^{2},$$

and to account for post-transitional effects as^50,51^

10 $${w}_{{{comp}}}=\frac{1}{2}\bar{B}{\left\{\frac{\partial u}{\partial z}-\frac{1}{2}\left[{\left(\frac{\partial u}{\partial x}\right)}^{2}{\left(\frac{\partial u}{\partial y}\right)}^{2}\right]\right\}}^{2}$$

**Fig. 4:**
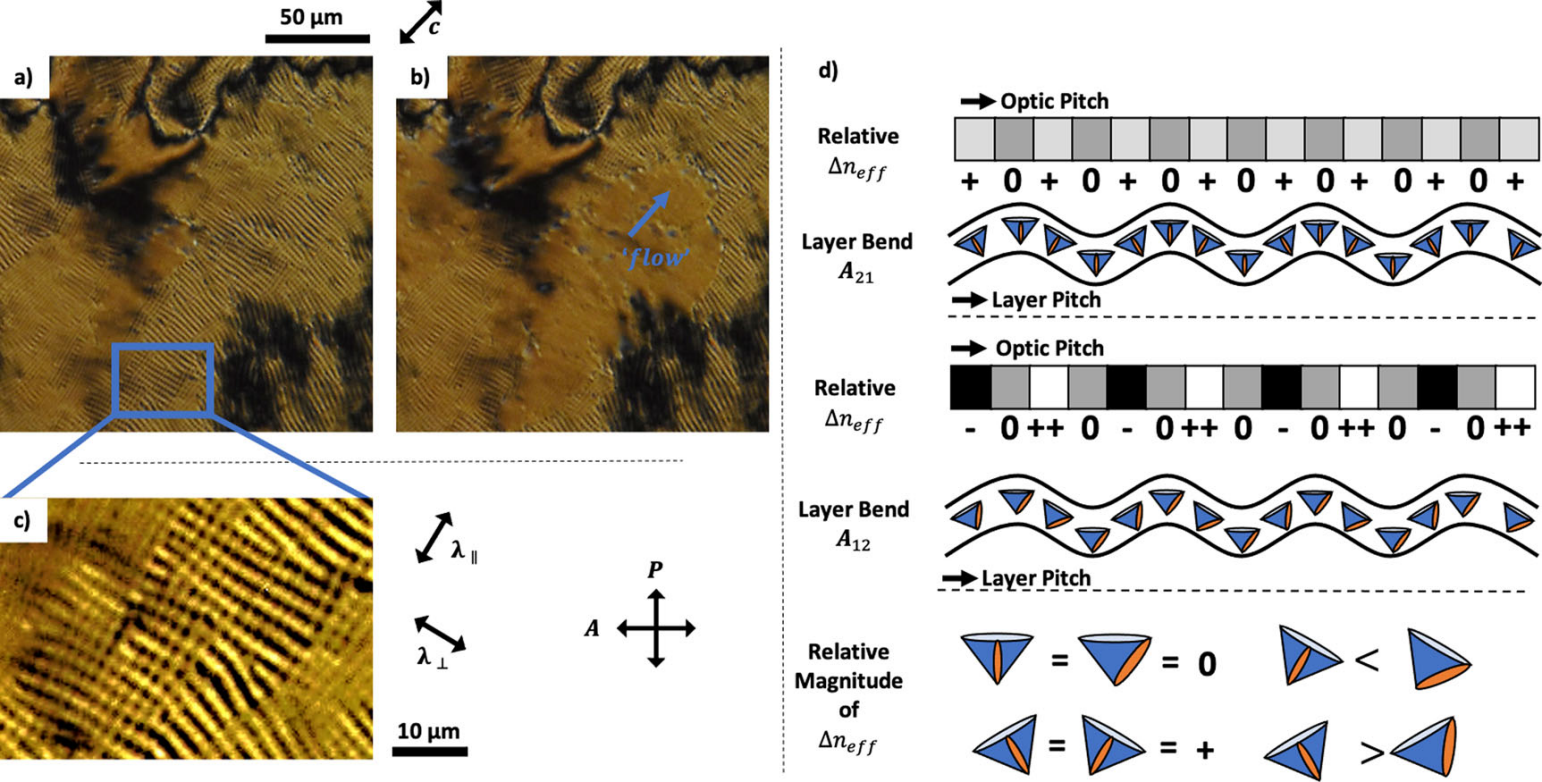
Polarising optical microscope images of layer undulations the surface-stabilised SmC(*) alignment in a simple homeotropic device. **a**–**c** A *d* = 25 ± 1 μm homeotropic cell with no grating alignment is filled with an 8.3% SCE13* mixture, resulting in a Schlieren texture. **a** The temperature is reduced at 1 °C/min to induce thermal undulations that are optically parallel to the **c**-director. **b** An example of a dislocation line of layer formation, separating regions of different numbers of smectic layers. **c** Magnification of a region showing double undulations, clearly showing a difference in their optical intensities and pitch. **d** A schematic demonstrating how the relativity intensity of the optical pitch relates to the layer pitch arising from A_12_ and A_21_ deformations in a homeotropic SmC(*). “0” indicates the expected intensity with no undulations, “−“ represents a decrease, “+” an increase, and “++” a significantly larger increase.

Here, the layer compressibility term $\bar{B}$ is assumed to be high and hence the amplitude of the layer undulations is small. This creates a cross-hatched pattern or texture, which annihilates behind a dislocation line that spreads over the sample on cooling, analogous to a Helfrich–Hurault type undulation^52,53^. Here, a double undulation is induced by first an undulation in the smectic layers deforming rather than forming new layer, and the second undulation by the periodic grating structure acting perpendicular to the direction of the first. The aligned **c**-director state is retained on further reduction to the temperature of the device, with continuous double undulation textures appearing and realigning behind waves of layer reorientation. It is worth noting that undulating textures are observed without the presence of gratings in our system. This phenomenon can be used to predict the relative magnitude of *A*_12_ and *A*_21_ for SCE13*, and thus the preferred **c**-director alignment on a surface-relief grating. The undulations within the smectic layers will form either parallel (${\lambda }_{\parallel ,{{Optical}}}$) or perpendicular (${\lambda }_{\perp ,{{Optical}}}$) to the **c**-director field. Such examples are shown in Fig. 4, including Fig. 4b which highlights the dissipation of the temporary undulations behind a moving boundary separating regions of a different number of smectic layers. Double undulations always form perpendicular to one another, and form in the instance where it becomes energetically favourable to undulate in a second direction rather than further increase the amplitude of the initial undulation and is related to the magnitude of *A*_12_ and *A*_21_. The optics of these undulations are investigated schematically in Fig. 4d, and relates the dark and bright fringes of the two undulations to changes in the relative birefringence caused by the director undulating through the layers. Figure 4d clearly highlights that the *A*_21_ undulation forms an optical undulating texture (${\lambda }_{\perp ,{{Optical}}}$) that is half the pitch of the actual physical undulation (${\lambda }_{\perp ,{{Layer}}}$). In the case of the *A*_12_ undulation, both the optical texture (${\lambda }_{\parallel ,{{Optical}}}$) and physical undulation (${\lambda }_{\parallel ,{{Layer}}}$) have an equivalent pitch, and has a larger change in relative magnitude than the *A*_21_ undulation so presents a stronger optical oscillation. Using Fig. 4 and understanding that the lower energy elastic distortion is more likely to occur, we measure that ${\lambda }_{\parallel ,{{Optical}}}=2.7\pm 0.2\,\mu {\mathrm {m}}$ and ${\lambda }_{\perp ,{{Optical}}}=2.2\pm 0.2\,\mu {\mathrm {m}}$, and deduce that ${\lambda }_{\parallel ,{{Layer}}} \sim 0.6{\times \lambda }_{\perp ,{{Layer}}}$, and so independently confirm that ${A}_{12} \, > \, {A}_{21}$. Hence, *A*_21_ undulations are predicted to form on surface-relief gratings. Measurements of these layer bending constants are rare, and so this analytical method shows relevance for easily establishing the expected **c**-director alignment on a surface-relief grating for a SmC(*) material. Interestingly, the addition of the surface grating exacerbates such undulations and define their texture. We propose that in such instances, the mechanism by which the layers reorient spontaneously on cooling is equivalent to the self-healing that occurs when the layers are disrupted by shock-induced flow.

**Fig. 5:**
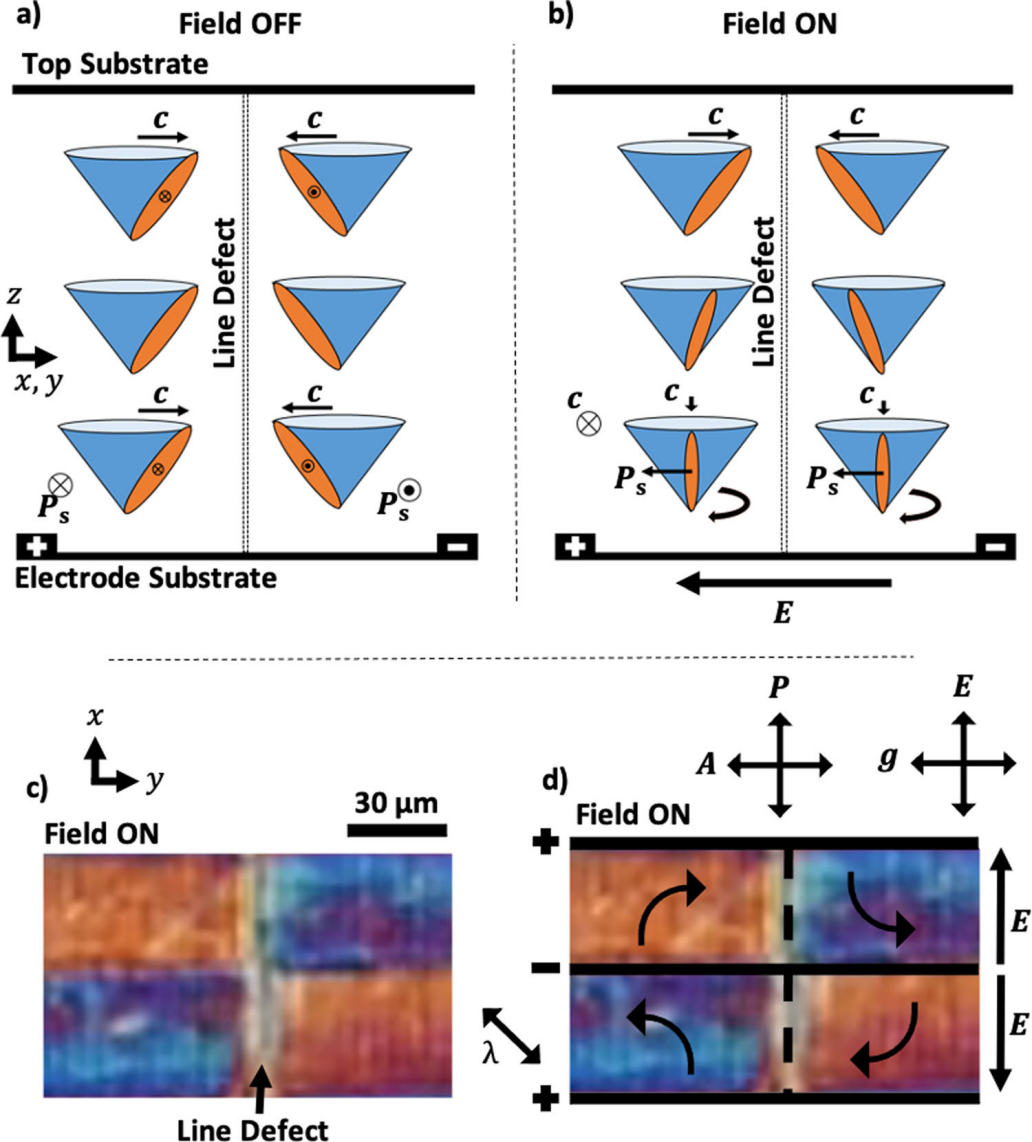
Defect origin in the VGA-FLC device. **a** and **b** A schematic diagram showing the origin of the defects that arise on a homeotropic grating. **a** Field OFF and **b** Field ON. As the **c**-director is initially at a 180° twist over the defect, an applied in-plane electric field will rotate the **c**-director in opposite directions. **c**, **d** Experimental polarising optical microscopy results showing how a full-wave plate is used to determine the direction of the **c**-director with a small electric field.

In addition to the Helfrich–Hurault-type smectic layer undulations shown in Fig. 4, defect lines that were not present in the SmA phase appear on cooling a few °C into the SmC*, which remain across the temperature range of the SmC* (Fig. 3l). Such domains could not be prevented by cooling slowly into the SmC* phase, even with an applied DC field. Additionally, these defects are not permanently located in the same position after heating and re-cooling into the SmC*, indicating that they are not solely due to unwanted containment effects from the processing of the gratings. The origin of these defects is that there are two possible directions of the **c**-director with the grating, at both ±90° (as ${{{{{\boldsymbol{c}}}}}}\ne -{{{{{\bf{c}}}}}}$). This is allowed by the symmetry of the sinusoidal grating and the *A*_21_ elastic deformation. If the gratings on opposing surfaces are parallel and the SmC* is surface stabilised, then both uniform **c**-director orientations are equally likely and so just two domains are found. To confirm the formation origin of this defect, an in-plane electric field was applied to the SmC(*) phase, with a magnitude lower than that required to fully switch the device. POM reveals that the **c**-director at the electrode surface rotates in opposite directions across the defect line. This is confirmed by the difference in retardation colour when observed with a full-wave plate at +45° to the crossed polarisers, as shown in Fig. 5a–d. This indicates that the **c**-director is oriented at 180° on either side of the defect line, and therefore twisting though the cell in opposing directions (clockwise and anti-clockwise). This scenario is described schematically in Fig. 5a, b, and optically in Fig. 5c, d. The defect lines cause a loss in optical contrast due to being bright. It is therefore important to remove them from the viewing area for an optimised device.

The aligned **c**-director state of the VGA-FLC is obtained due to the low-amplitude and relatively high-pitch of the sinusoidal-like gratings. Without inclusion of a grating, the limiting alignment is a Schlieren texture where there is no preferred alignment to the **c**-director. The effect of increasing the amplitude of the surface-relief gratings is unlikely to increase smectic layer undulations, since this will be set by the compressibility of the smectic layers: higher amplitude gratings merely result in regions of mismatch between the surface anchoring and the grating wall. Eventually, when the grating amplitude is too large, it increases the stability of the double undulating state, described by the carpet-like textures, which does not dissipate on cooling. It is understood that the penetration length of an induced undulation in the SmA phase will change depending on the properties of the grating, and is generally much larger than the wavelength of the undulation at the interface^46^. Increasing or decreasing the pitch of such gratings will therefore likely alter the effects of the self-healing process in the SmC* phase. Ultimately a balance must be struck relating how strongly the **c**-director is forced to lie parallel with the grating, and how much disruption is allowed to the smectic layers from the grating amplitude, when optimising device behaviour. This is an interesting topic for further study, as the grating structure shown in this paper is not optimised.

### Grating induced self-healing of the FLC *c*- and *a*-directors

**Fig. 6:**
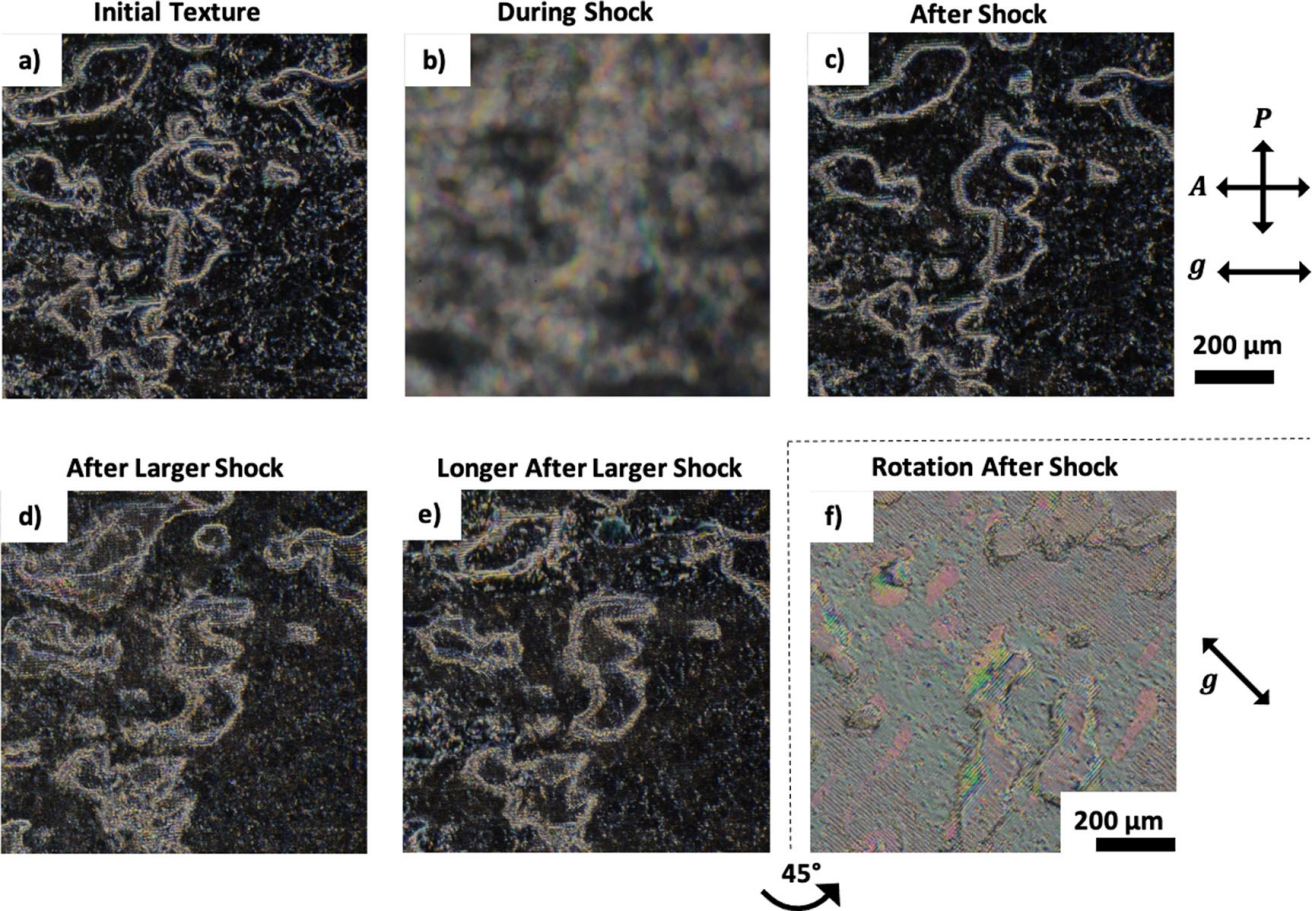
Self-healing of the VGA-FLC. **a**–**f** Shock-induced flow of VGA-FLC Device 1 (Table 1) at *T* = 20 °C. **a** The dark texture observed with polarising optical microscopy, where **g** is aligned with the polarisers. **b** The application of a low-pressure shock induces flow to the LC in the device. **c** The alignment of the bulk is unaffected by the application of a small pressure (4.0 ± 0.7 N). **d** A large shock (18±1 N) induces small A_12_ undulations into the texture, which can only be observed as a slight increase in transmission due to a double-undulating texture b**e**ing induced. **e** The texture 30 min after the initial shock remains almost unchanged, but some dissipation of the double-undulating texture has occurred. **f** 45° rotation of the device reveals the metastable texture of the undulations induced by shock-induced flow, which highlight undulations induced perpendicular to **g**. Importantly, the **c**-director is still well aligned after shock-induced flow.

The smectic layers in this device geometry were found to be considerably more resistant to shock-induced flow than conventional SSFLC, as shown in Fig. 6. The homeotropic geometry itself is less sensitive to shock: any mechanical force applied through pressing the glass plates causes compression and **c**-director rotation rather than layer deformation, schematically shown in Fig. 1j–o. This is contrary to what happens in the planar aligned SSFLC, where changes to the layer tilt and associated zig–zag defects are induced by small applied pressures, and high degrees of shock lead to the induction of homeotropic aligned layers^8,54^. It has been discussed that the sinusoidal relief grating strongly orients the **c**-director to lie in a particular direction, shown in Fig. 3, resulting in a uniform SmC(*) texture. On a mechanical shock, the device goes bright due to significant **c**-director disruption, and a small amount of layer deformation and compression, seen in Fig. 6b. Following the applied shock, a similar effect to the Helfrich–Hurault-type undulations is observed, whereby a double undulating texture is quickly adopted, perceived through the optical texture. It is then observed to slowly dissipate, or self-heal, in a similar fashion to that observed on device cooling, whereby the homeotropic **a**-director and the **c**-director once again realign with the surface-relief grating, removing the undulating texture and restoring the dark texture to the device. It should be noted that in the double undulating texture the **c**-director is still aligned with the grating, but the increased undulations reduce the optical contrast. For example, on rotation between crossed polarisers this undulating texture will appear brightest at 45°. Without surface-relief gratings, a subtle undulating texture is induced into the smectic layers following a mechanical shock, aligning with the direction of the flow to the **c**-director. It is the combination of both the physical shape of the surface-relief gratings and the interactions between the LC and the alignment layer that results in the observed self-healing mechanism.

### Application of in-plane fields to FLC device

**Fig. 7:**
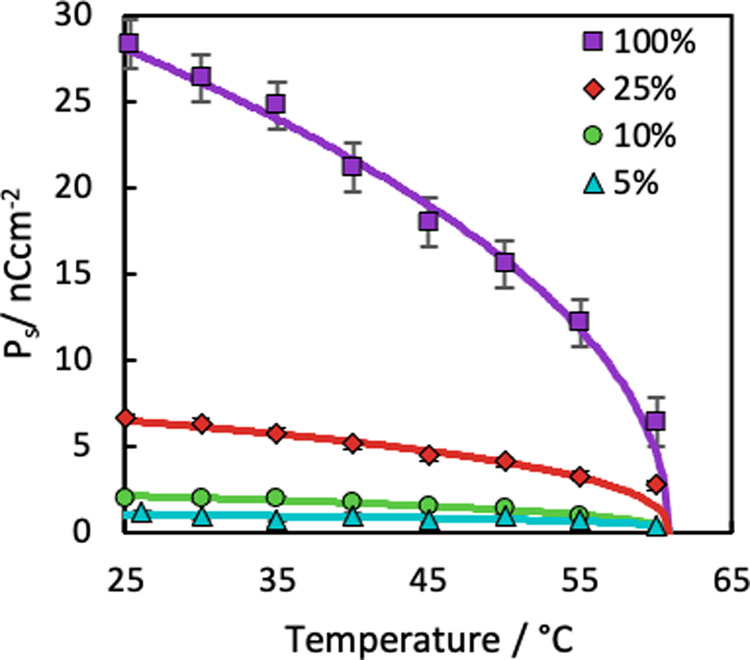
Spontaneous polarisation measurements of mixtures of SCE13* and SCE13-R. Measurements were taken using the current-reversal technique using a 30 V, 113 Hz triangular waveform on planar-aligned devices. The experimental error bars were calculated from the precision of the area measurement for the current-reversal technique (trapezium rule), together with the uncertainties of measuring mass for the mixtures. The lines are fits to Eq. (8). The legend refers to the ratio of SCE13* to SCE13-R; that is, “5%” indicates that the mixture contains 5% SCE13* to 95% SCE13-R.

In-plane electric fields were applied using IDEs on one surface of the VGA-FLC device, as shown schematically in Fig. 1a–e, i–j. Table 1 summarises the cell gaps, electrode spacing, and spontaneous polarisation of the mixtures of SCE13* and SCE13-R used for the response time measurements, and Fig. 7 summarises the spontaneous polarisation measurements over the temperature range of the SmC* phase. No helicity was observed in the devices due to satisfying Eq. (1), and sufficient anchoring strength provided by the gratings from Eq. (3). We consider the expected director change through the cell (*z*-direction) to be proportional to the magnitude of the electric field, which follows^55^

11 $${{{{{{\boldsymbol{E}}}}}}}_{{{{{{\boldsymbol{z}}}}}}} \sim \frac{{{{{{\boldsymbol{V}}}}}}}{l}{\cos }\left(\frac{\pi y}{l+w}\right){\exp }\left(-\frac{\pi z}{l+w}\right)$$

where *w* and *l* are the size of the width of the electrode and spacing between them, respectively, and ***V*** is the applied electric voltage over the IDEs. Importantly, it should be understood that in reality the electric field is periodic following the grating vector of the IDEs, and decays exponentially through the bulk. Most treatments assume that for sufficiently low *d* and *w* that ***E*** is uniform across the cell plane and can be given approximately by

12 $${{{{{\boldsymbol{E}}}}}}\approx \frac{{{{{{\boldsymbol{V}}}}}}.d}{l}$$

**Table 1:** Physical properties of three VGA-FLC devices.

Device #	${{{{{\boldsymbol{|}}}}}}{{{{{{\bf{P}}}}}}}_{{{{{{\bf{s}}}}}}}{{{{{\boldsymbol{|}}}}}}$ (nC cm^−2^) (30 °C)	*d* (µm)	${A}_{{{\mathrm {pp}}}}$ (µm)	Electrode width (µm)	Electrode gap (µm)
1	$0.95\pm 0.05$	$30\pm 2$	$0.23\pm 0.08$	$7.9\pm 0.7$	$22.1\pm 0.7$
2	$2\pm 0.1$	$12\pm 2$	$0.26\pm 0.07$	$7.9\pm 0.7$	$22.1\pm 0.7$
3	$6.4\pm 0.3$	$8.3\pm 0.7$	$0.26\pm 0.07$	$4.0\pm 0.1$	$8.0\pm 0.1$

On the application of a sinusoidal waveform of low frequency (*f* ⁓ 0.1 Hz) and voltage >10 V, the optical transmission oscillates between bright and dark states repeatedly as the **c**-director reorients with the applied field. At higher frequencies (between 1 and 10 kHz) the optical transmission does not change visibly, nor through use of a photodiode. This indicates that the **c**-director remains close to or at its equilibrium position aligned perpendicular to **g**, or OFF state, and so the device remains dark. See the Supplementary Information for further information regarding the electro-optic response of the device (Supplementary Figs. 1–3). This is an interesting result of the quickly oscillating sign of the electric field changing faster than the response time of the material, and so resulting with the **c**-director remaining aligned with **g**. Accordingly, the device should be operated utilising DC fields, as is appropriate for a FLC in an LCoS device. Either side of an electrode, the SmC(*) is exposed to the same magnitude but opposite sign of electric field, shown in Fig. 1j. Therefore, the **c**-director twists in opposite directions (either clockwise or anti-clockwise) on either side of the IDE throughout the device. Both conditions appear optically identical, and opposing twists can be distinguished through the use of a full wave plate, shown in Fig. 5. This type of arrangement helps ensure that the viewing angle of the device is high, despite the high tilt of the opposing twisted nematic like domains. When the sign of the field is reversed about the electrode, the direction of the induced helical twist of the **c**-director swaps handedness. In doing so, the optics temporarily return to a dark state where there is no twist through the cell (the OFF state). The total twist angle of the **c**-director as a function of applied voltage is shown schematically in Fig. 2. When the field is too low, the total twist observed is <90°; fields above the optimum causes the **c**-director to over-twist past the 90° condition. Importantly, the **c**-director at the opposing substrate remains strongly anchored and is not observed to change orientation. It should be noted that there is no optical response in the racemic material device with these magnitude electric fields, indicating that the result is indeed a ferroelectric response, and there is no or minimal flexoelectric switching arising from the undulating geometry.

**Fig. 8:**
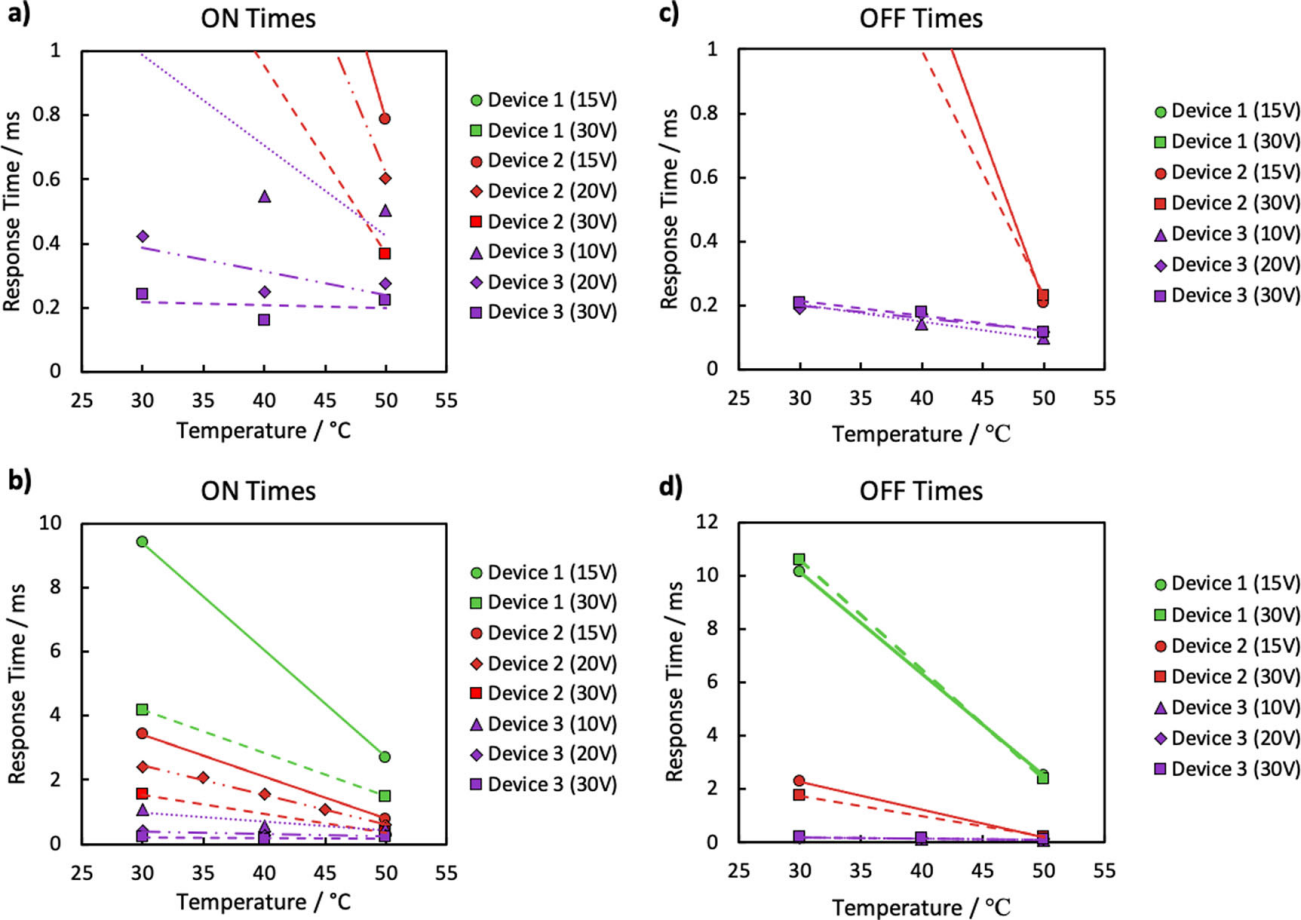
Response times for prototype VGA-FLC devices. The response times for three VGA-FLC devices (Table 1) versus temperature for various applied voltages. Measurements were made with ON and OFF pulses of the same but opposite magnitude voltage. **a**, **b** ON times for Device 1 and **c**, **d** OFF times. All lines of best fit are guides for the eye representing Eq. (5), however, are not necessarily expected to be linear due to the rotational viscosity also showing non-linear temperature dependence. Further details are given in Supplementary Information.

Response times were measured by applying asymmetric square waves to switch the **c**-director 90° through the cell. OFF times were measured by allowing the **c**-director to relax to its OFF position from its ON state, as well as by applying a restorative pulse to bring the **c**-director back into the OFF-state alignment. Figure 8 shows the ON and OFF times for the three devices. Response times are calculated from 10% to 90% relative transmission, and are shown to be sub-millisecond under certain conditions. As an example, the SSFLC device demonstrates ON and OFF times are equally fast and must be latched with an electric pulse due to the inherent bistability of the two states. For the VGA-FLC, the ***c***-director switching time in response to coupling between the spontaneous polarisation ${{{{{{\mathbf{P}}}}}}}_{{\mathrm {S}}}$ and applied field **E** is given by^38,56,57^

13 $${\tau }_{{{ON}}(\pm V)}={\tau }_{{{FIELD}}}=\frac{{\gamma }_{\varPhi }}{{{{{{{\mathbf{P}}}}}}}_{{s}}{{{{{\mathbf{E}}}}}}{{{{{\boldsymbol{-}}}}}}{\left(\frac{{{{{{\boldsymbol{\pi }}}}}}}{{{{{{\boldsymbol{d}}}}}}}\right)}^{{{{{{\bf{2}}}}}}}{{{{{{\boldsymbol{B}}}}}}}_{{{{{{\bf{3}}}}}}}} \sim \frac{{\gamma }_{\varPhi }}{{{{{{{\mathbf{P}}}}}}}_{{s}}{{{{{\mathbf{E}}}}}}},$$

where ${\gamma }_{\varPhi }$ is the rotational viscosity describing reorientation of the **c-**director, which is often approximated as ${\gamma }_{1}{{\sin }}^{2}{\theta }_{{\mathrm {C}}}$, and assuming that the elastic terms are negligible compared to the electric torque. The **c**-director at the opposing surface to the IDEs is assumed to be anchored and not reorient, which is restricted to the bulk of the device, as described by Fig. 2. On removal of the field, the twisted ***c***-director profile relaxes to the original uniform state through the influence of the twist elastic constant *B*_3_. Through analogy to the nematic response time τ_OFF_^27^, the elastically driven relaxation time for the FLC can be approximated to

14 $${{\tau }_{{{OFF}}(0\,{{V}})}=\tau }_{{{{ELASTIC}}}} \sim \frac{{\gamma }_{\varPhi }{d}^{2}}{{\pi }^{2}{B}_{3}},$$

where the viscoelastic contribution is a result of the **c**-director twist (*B*_3_) elastic constant of a SmC(*). For the VGA-FLC used here, the device may be switched OFF either through the spontaneous elastic relaxation described by Eq. (14), or by reversing the sign of the applied field and driving the ***c***-director profile back towards the undistorted state (as ${{{{{\boldsymbol{c}}}}}}\ne -{{{{{\boldsymbol{c}}}}}}{{{{{\boldsymbol{)}}}}}}{{{{{\boldsymbol{:}}}}}}$

15 $${\tau }_{{{{OFF}}}(\mp V)}={\tau }_{{{{FIELD}}}} \sim \frac{{\gamma }_{\varPhi }}{{{{{{{\mathbf{P}}}}}}}_{{{s}}}{{{{{\mathbf{E}}}}}}{{{{{\boldsymbol{+}}}}}}{\left(\frac{{{{{{\boldsymbol{\pi }}}}}}}{{{{{{\boldsymbol{d}}}}}}}\right)}^{{{{{{\bf{2}}}}}}}{{{{{{\boldsymbol{B}}}}}}}_{{{{{{\bf{3}}}}}}}}\approx \frac{{\gamma }_{\varPhi }}{{{{{{{\mathbf{P}}}}}}}_{{{s}}}{{{{{\mathbf{E}}}}}}},$$

In this latter case both the ON and OFF response times are approximately equal and appreciably faster, and the elastic terms are often assumed to be negligible compared to the electric torque.

ON and OFF times were faster for smaller cell gaps due to a higher effective field at the same applied voltage, and with mixtures with a larger spontaneous polarisation. Higher temperatures at 50 °C produce faster response times than at 30 °C due to a decreased rotational viscosity, *γ*_*φ*_, of the FLC^56,58^. Sub-millisecond optical switching times were recorded for Device 3 at temperatures of 30 °C. Response times of ${\tau }_{{{{ON}}}(\pm V)}=242\pm 5\,{\mu {\mathrm {s}}}$ and ${\tau }_{{{{OFF}}}(\mp V)}=208\pm 6\,{\mu {\mathrm {s}}}$ were achieved with an applied voltage of 30 V at 30 °C, with the difference potentially caused by the opposite elastic contribution between Eqs. (13) and (15). Additional times over a range of temperatures and applied voltages for three devices with different **P**_s_ mixtures, cell gaps and IDE electrode spacing’s are given in Fig. 8, where the key properties of the devices are summarised in Table 1. It was seen that the response times increase on decreasing temperature despite an increase in **P**_s_, which is a property found with most FLCs^58^. Without an applied restorative field, the **c**-director relaxes back to its OFF state significantly slower than with a restorative pulse, although should be noted that sub-millisecond times are still observed, and so can be considered as an option if required for a particular application. This elastically driven OFF time can be significantly slower than for conventional FLC modes, both because the cell gap d is typically higher and because the **c**-director twist elastic constant *B*_3_ is low^59^. The pulsed OFF times are of a similar magnitude as the ON times in this VGA-FLC geometry, as expected from Eq. (13), and it is anticipated that in practice the device will be electrically driven to both ON and OFF states to maximise response speed. The effect of changing the amplitude, pitch and surface treatment on the switching mechanism and times of the device has not been studied in detail, however it is not expected such changes would affect the electro-optic response, rather than modify the initial alignment quality of the FLC.

## Discussion

As discussed previously, the literature includes various innovative options for the alignment and electrical addressing of FLC electro-optic devices. A key advantage of these modes over nematic based solutions is the inherently faster optical response times, usually targeting responses below 1 ms to enable 240 Hz frame video refresh rates and frame sequential colour^60^. FLC polar switching allows devices to be driven electrically in both directions, ON–OFF and OFF–ON, rather than have one of the responses driven solely by the slower elastic relaxation as with conventional nematics. We have shown that the VGA-FLC geometry can achieve these required response times, in addition to demonstrating an alignment geometry that suppresses the helix of the FLC and enables a self-healing of disturbed smectic layers after a mechanical shock, which has not been shown previously.

Significant improvements can be made to optimise the device. For example, further decease of the optical response times is readily achieved using an improved choice of FLC material with a naturally larger **P**_s_ and pitch. The material SCE13* used in these prototype devices due to availability has a **P**_s_ of $26\pm 1\,{{\mathrm {nC}}\,{\mathrm {c}}}{{\mathrm {m}}}^{-2}$ at 30 °C, with a helical pitch of *P* = 10–15 μm, and so mixtures were made with the racemic mixture to reduce the pitch, resulting in also lowering the **P**_s_. SCE13* was designed for use in SSFLC, and subsequently, materials with much higher **P**_s_ ($> 100\,{{\mathrm {nC}}\,{\mathrm {c}}}{{\mathrm {m}}}^{-2}$) have been produced. Such mixtures will directly result in both faster ON and OFF times due to Eq. (5), while a larger pitch is important to avoid unwanted helicity forming through the device, and so satisfying Eq. (1) even with the relatively high cell spacings allowed by this mode. In practice, a different mixture would be chosen to combine the higher **P**_s_ and helical pitch P with a higher SmC cone angle, *θ*_C_, and birefringence, Δ*n*. This route will lead to significant further improvements to the response times. For the 90° twist ON state to appear white, the Gooch–Tarry equation must be satisfied for a wavelength of 550 nm as introduced in Eq. (1). For the homeotropic SmC(*) used throughout the experiment, the resultant birefringence of a surface stabilised state is obtained from:

16 $${n}_{{{{eff}}}}\left(\theta \right)=\frac{{n}_{{\mathrm {o}}}{n}_{{\mathrm {e}}}}{\sqrt{{{n}_{{\mathrm {o}}}}^{2}{{cos }}^{2}{\theta }_{{\mathrm {C}}}+{{n}_{{\mathrm {e}}}}^{2}{{sin }}^{2}{\theta }_{{\mathrm {C}}}}},$$

where *n*_e_ and ${n}_{{\mathrm {o}}}$ are the extraordinary and ordinary refractive indices, respectively, and ${\theta }_{{\mathrm {C}}}$ the cone angle. The biaxiality of the material is assumed to be negligible. Using *n*_e_(30 °C) = 1.67, ${n}_{{\mathrm {o}}}$(30 °C) = 1.49 and ${\theta }_{{\mathrm {C}}}$(30 °C) = 22.3° for SCE13*^61,62^, a resultant birefringence for the homeotropic FLC device of ${\triangle n}_{{{\mathrm {eff}}}}=0.028$ is obtained. Therefore, a relatively high cell gap of about 20 µm is required for this device to optically appear white, or 10 µm for a reflective device. As such, a material with a higher birefringence and cone angle would allow the cell gap of the optimum device to be reduced while satisfying the Gooch–Tarry equation for white light transmission, resulting in faster response times. For example, the SmC* phase of the recent mixture W107^63^ has a cone angle of *θ*_C_ ≈ 39°, Δ*n* ≈ 0.12 and **P**_s_ of 150 nC cm^−2^. These optical properties give ${\triangle n}_{{{\mathrm {eff}}}}\approx 0.051$, allowing the cell gap to be reduced to 55% that of SCE13, which, if the viscosity is assumed to be the same, would correspond to the electrically driven switching speed being increased by over a factor of 2. Moreover, the switching torque would be increased as given by Eq. (13) and so would be increased by a further factor of 4. Further reduction of the switching speeds will result from use of single polariser reflective TN devices (such as those used for LCoS) by using a 75° twist through the device with polarisers at −7.5°, further reducing driven switching speeds by 50% compared to a 90° twist, and requiring a 20% decrease in cell gap^27^. Introducing such modifications suggests that it is realistic to achieve response times in the order of 10’s of microseconds.

One outstanding complication of the geometry is the defect lines caused by the two possible orientations of the **c**-director relative to the grating vector, which result in disruptive bright defect lines. Despite this, large areas of defect free alignment are shown, and importantly display a resistance to shock. For practical applications it is important to remove these disclination lines. This may be realised in two ways. First is the introduction of a second set of IDEs on the opposing substrate. On cooling into the SmC(*), sufficiently high in-plane fields on both sides should induce orientation in the grating in the same direction, directing the disclinations to lie above the electrodes, an area that is not used optically. On removal of the field, the **c**-director will relax to lie along the grating in just one orientation between electrodes. This additional complexity is not however desirable for the manufacturing process or minimising cost. Therefore secondly it may also be possible to remove the defects by introducing a blazed grating geometry to preferably orient the **c**-director in only one direction, removing defects altogether in the SmC(*) phase^28^. Ultimately the removal of these defects relies on a method to orient the **c**-director on cooling from the SmA phase into only one alignment preference coinciding with the lowest state. Once well aligned in the SmC(*) phase, defects should not arise following a mechanical shock due to the surface anchoring, nor following prolonged electrical addressing, again due to the preferred **c**-director orientation.

In closing, an FLC electro-optic device has been reported using a shock-resistant homeotropic geometry, named the VGA-FLC. The combination of surface-relief gratings treated with octyltrichlorosilane and IDEs for in-plane electrical addressing has shown desirable SmC(*) alignment and electro-optic switching for use in high speed displays and devices. The surface-relief gratings provide an alignment preference for the **c**-director of the surface stabilised SmC(*), while the homeotropic surface treatment helps maintain the layer director to lie uniformly parallel to the substrate normal. Following a mechanical shock, the layers return to their homeotropic alignment following layer compression, and then the induced undulations return to an aligned **c**-director state determined by the surface-relief grating, which is seen optically in the device as self-healing. Such a mechanism has not been reported before for a SmC(*). Sub-millisecond response times are obtained for a prototype device at 30 °C, with ${{{{{{\rm{\tau }}}}}}}_{{{{{{\rm{ON}}}}}}(\pm {{{{{\rm{V}}}}}})}=242\pm 5{{\mu {\mathrm {s}}}}$ and ${{{{{{\rm{\tau }}}}}}}_{{{{{{\rm{OFF}}}}}}(\mp {{{{{\rm{V}}}}}})}=208\pm 6{{{{{\rm{\mu s}}}}}}$ with an applied voltage of 30 V. Such times are expected to reduce further on device optimisation, achieved by changing both the material properties of the FLC and some geometries of the device, both beyond the scope of this work. Careful selection of the material properties, such as cone angle and birefringence, would enable smaller cell gaps to be used for the device and so further decreasing the observed response times. Removal of line defects inherent to the geometry is required, which can be realised by optimising the profile of the surface-relief grating to allow only one preferred alignment orientation.

## Methods

### Surface-relief grating fabrication

**Fig. 9:**
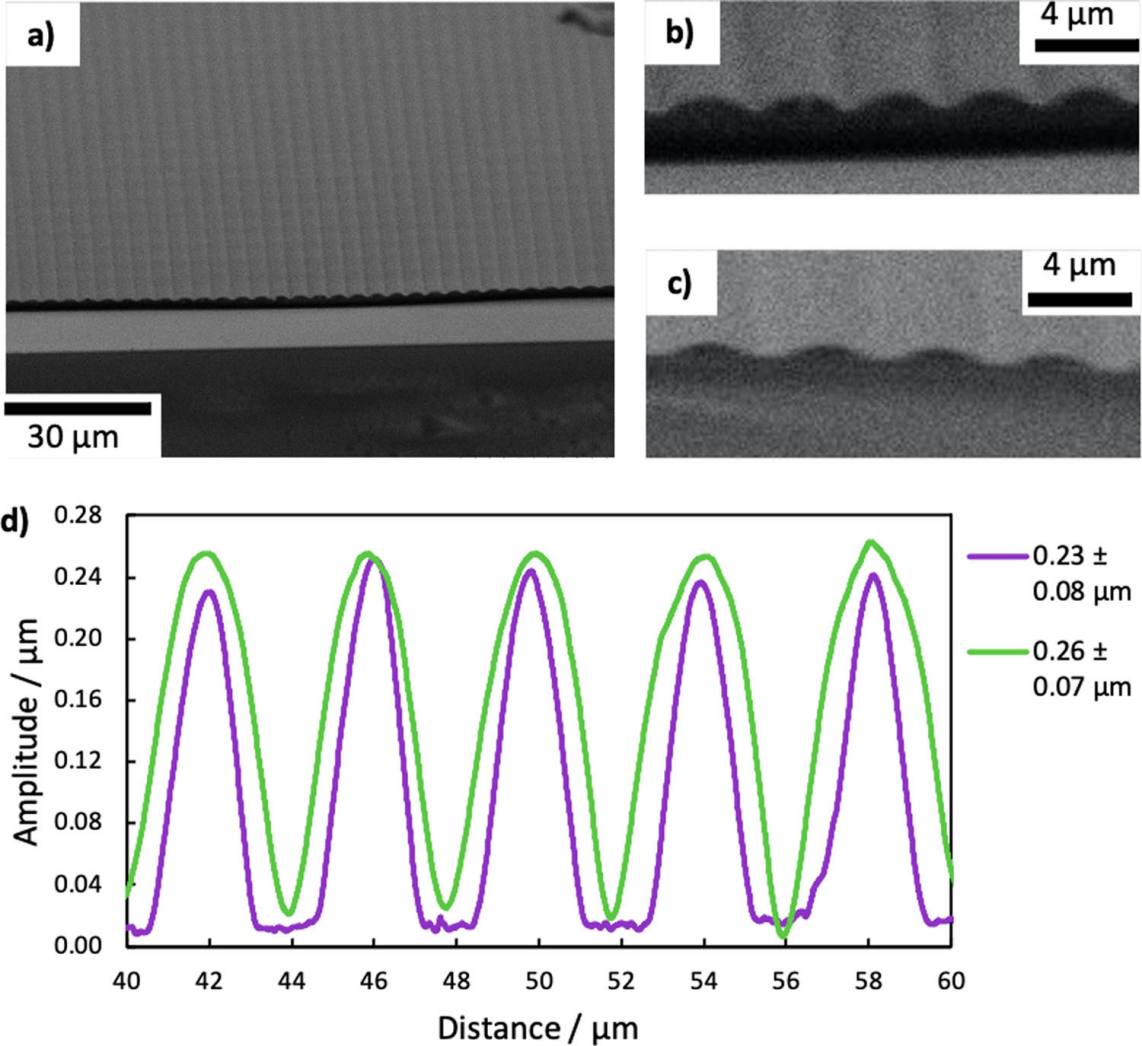
Fabrication of gratings for the VGA-FLC device. SEM images of SU-8 surface relief gratings. **a**, **b** a $2.32\pm 0.05$ μm film thickness, with a dose of $500\,{{\mathrm {mJ}}\,{\mathrm {c}}}{{\mathrm {m}}}^{2}$, resulting in a peak-to-peak amplitude of $0.77\pm 0.02$ μm with 1.6 μm offset. **c** A 0.66 ± 0.09 μm film thickness, with a dose of $1000\,{\mathrm {mJ}}\,{\mathrm {c}}{{\mathrm {m}}}^{2}$, resulting in a peak-to-peak amplitude of $0.59\pm 0.05\,{\mathrm {\mu}} {\mathrm {m}}$ with no offset. **d** The surface profiles of the two master gratings used in the VGA-FLC devices. These masters were created using a 30% dilution of SU-8 2025, and exposed using a 2 µm, 375 nm laser in the direct-write laser lithography system. Final SU-8 layer thickness: $0.84$ ± $0.05$ μm. Grating peak-to-peak amplitudes: Purple line: $0.23\pm 0.08\,{\mathrm {\mu}} {\mathrm {m}}$, using a dose of $550\,{\mathrm {mJ}}\,{\mathrm {c}}{{\mathrm {m}}}^{-2}$, and green line: 0.26 ± 0.07 μm, using a dose of $1200\,{\mathrm {mJ}}\,{\mathrm {c}}{{\mathrm {m}}}^{-2}$.

A surface-relief grating master was fabricated using the permanent epoxy negative photoresist SU-8 2025 (MicroChem Corp.) on ITO glass (Displaydata Ltd.) with the adhesion promoter Omnicoat™ (MicroChem Corp.). The SU-8 2025 was diluted to be 30% by weight in cyclopentanone (Sigma-Aldrich) to create a $0.8\pm 0.1\,\mu {\mathrm {m}}$-thick film on spin-coating. The SU-8 solution was spun at $500\,{\mathrm {{rpm}}}$ for $10\,{\mathrm {s}}$ at $100\,{{\mathrm {rpm}}}/{\mathrm {s}}$, then $3000$ rpm for 40 s at 255 rpm/s, and soft baked at 30 °C for 30 min. The resist was patterned using a 2 μm diameter, 375 nm wavelength UV laser using a MicroWriter ML2 (Durham Magneto Optics Ltd.) direct laser writer. Surface-relief gratings were written using $550$ and $1200\,{\mathrm {mJ}}\,{\mathrm {c}}{{\mathrm {m}}}^{-2}$ doses of UV, by writing 1 cm long lines of width 2 μm and pitch 4 μm. After exposure, the sample was post exposure baked at 50 °C for 30 min, and then developed by in propylene glycol monomethyl ether acetate (Sigma-Aldrich) to remove the remaining uncured resist. The master was hard baked at 180 °C for 2 h. This process produced gratings with peak-to-peak amplitudes of ~0.2–0.3 μm and with a 4 μm pitch. Benchtop electron microscopy (Hitachi Ltd., TM3030Plus) was used to image surface-relief gratings, where an example of a grating is shown in Fig. 9a–c. The profile of the SU-8 master gratings used I the devices presented here was measured using a Dektak XT^®^ Surface Profiler (Bruker), and is presented in Fig. 9d.

### Electrode (IDE) fabrication

Interdigitated electrodes (IDEs) were fabricated in-house from ITO glass with a 100 nm-thick layer of ITO using the positive tone Shipley Microposit S1813 (Microchem Corp.), a photomask (Nanofilm Inc.) with the IPS electrode design (made in-house) on a mask aligner (Model 200, OAI) and acid etching. Glass was cut and coated using a spin-coater (WS650MZ, Laurell Tech. Corp.) at 400 rpm for 3 s at 100 rpm/s, 1000 rpm for 3 s at 100 rpm/s, and 4000 rpm for 60 s at 355 rpm/s. This was then soft baked at 115 °C for 60 s. This photoresist-coated ITO substrate was exposed using a mask aligner in soft contact for 9.3 s. The exposed photoresist was then developed in a 2% tetramethylammonium hydroxide and 98% deionised water mix, with trace amounts of Decon-90 (around 2 drops from a pipette per 100 mL of mix) for 30 s, and hard baked at 150 °C for 10 min, to prevent delamination whilst submerged for the acid etch. The patterned substrate was acid etched in a 1:1:1 solution of deionised water, glycerol, and hydrochloric acid (37% by weight in water) for 1 h 15 min at 30 °C. The photoresist was removed with acetone, and the IPS electrodes cut to size and thoroughly cleaned. The final electrodes had an electrode spacing of $22.1\pm 0.7$ µm, electrode width of $7.9\pm 0.7$ µm, and overall pitch of 60 µm, with an active electrode area of 0.5 mm^2^. Electrodes with $8\pm 0.1$ µm gap and $4\pm 0.1$ µm were also used, provided by Merck Chemicals Ltd.

### Embossing gratings onto IDEs

The surface-relief gratings were copied from the master and embossed into a photopolymer film onto both IDEs and plain glass substrates. This replication technique allows quick processing of identical gratings and helps remove unwanted offset of the resin beneath the grating structure, reducing the dielectric loss of the fields over the liquid crystal in the cell. The stamp was made in a mixture of 45% 1,6-hexanediol diacrylate (HDDA), 15% trimethylolpropane triacrylate (TMPTA), 40% Actilane420 and 1% of the photoinitiator Genocure LTM onto the master grating and topped with 175 μm-thick PET film. This was cured for 10 min in a UV EPROM eraser to solidify the resin. An adhesion promoter was prepared and deposited onto cleaned IPS electrodes and 1 × 1 cm^2^ pieces of cut-glass slides (1.2 mm thick), by evaporating at ambient temperature for 10 min and then baked at 130 °C for 60 s. The photopolymer PP2^32^ was applied between the adhesion promoter-coated substrates and the stamp. This was embossed at 6.5 mm s^−1^ and pressure of 4 bar, which produces a low offset in the photopolymer. This was cured for 2 min 30 s in the UV EPROM eraser. The PET stamp was peeled off, and the embossed grating remained on the glass substrates.

### Vapour phase silane deposition

Homeotropic or vertical alignment was applied to the grating substrates using the vapour phase deposition of octyltrichlorosilane (ABCR GmbH & Co.KG). 20 μL of silane was heated to 80 °C and allowed to flow through and deposit onto the surface-relief grating substrates for 5 min. Following exposure, the silane is baked at 180 °C for 1 h to chemically bind the silane to the substrate. This produces a uniform monolayer of silane^32^, and importantly gives a good homeotropic alignment to the FLC SCE13(*) over its liquid crystalline phases.

### Device construction and characterisation

Test cells were constructed using two grating substrates, one with IDEs and one without. Ten-micron diameter dry borosilicate glass microspheres (Duke Standards) were mixed with UV curable glue (NOA68, Norland Products Inc.) and used to provide a uniform cell gap to the device. Substrates were aligned keeping the gratings parallel, clamped and cured in a UV lightbox (LP6, Dezac Group Ltd.). Cell gaps were measured using reflection spectrometry (Olympus BH2-UMA microscope) before filling. Devices were capillary filled at 120 °C with mixtures of the FLC SCE13(*). SCE13* undergoes the following phase transitions:

$${{{Cr}}}\to < -20^\circ {{{{{\rm{C}}}}}}\to {{{Sm}}}{{\mathrm {C}}}^{\ast }\to 60.8\,^\circ {{{{{\rm{C}}}}}}\to {{{SmA}}}\to 86.3\,^\circ {{{{{\rm{C}}}}}}\to {N}^{\ast }\to 100.8\,^\circ {{{{{\rm{C}}}}}}\to I$$

SCE13* has a helical pitch *P* = 10–15 μm over the majority of the temperature range of the SmC* phase. To ensure no helical twist of the LC director through the device, the FLC helical pitch was increased by mixing the chiral and racemic mixtures of the same liquid crystal, SCE13* and SCE13-R. This method also reduces the magnitude of the spontaneous polarisation **P**_s_, but it allows the necessary pitch to be achieved without a change in the refractive indices or phase transitions of the FLC. Three mixtures were prepared at 1:3, 1:10 and 1:20 ratios of SCE13* to SCE13-R, respectively, increasing the chiral pitches to be >40, 100 and 200 µm, respectively. This results in a concomitant reduction in the spontaneous polarisation |${{{{{{\bf{P}}}}}}}_{{{{{{\bf{S}}}}}}}{{{{{\boldsymbol{|}}}}}}$ at 30 °C from ${{{{{{\mathbf{P}}}}}}}_{{{s}}(100 \% )}=26.4\,{{{{{\rm{nC}}}}}}\,{{{{{\rm{c}}}}}}{{{{{{\rm{m}}}}}}}^{-2}$, to, ${{{{{{\mathbf{P}}}}}}}_{{{s}}(25 \% )}=6.3\,{{{{{\rm{nC}}}}}}\,{{{{{\rm{c}}}}}}{{{{{{\rm{m}}}}}}}^{-2}$, ${{{{{{\mathbf{P}}}}}}}_{{{s}}(10 \% )}=2.0\,{{{{{\rm{nC}}}}}}\,{{{{{\rm{c}}}}}}{{{{{{\rm{m}}}}}}}^{-2}$ and ${{{{{{\mathbf{P}}}}}}}_{{{s}}(5 \% )}=0.95\,{{{{{\rm{nC}}}}}}\,{{{{{\rm{c}}}}}}{{{{{{\rm{m}}}}}}}^{-2}$ respectively. The spontaneous polarisations of pure SCE13* and its mixtures are presented in Fig. 7*,* where the lines of best fit were calculated using:

17 $${{{{{{\mathbf{P}}}}}}}_{{{s}}}={P}_{0}{({T}_{{\mathrm {C}}}-T)}^{\beta },$$

where *P*_0_ is a material or mixture constant, and *β* is an expression derived from Landau theory^2^. The FLC mixtures were capillary filled into three different devices with cell gaps, *d*, of 8.3, 12 and 30 µm, respectively, and gratings with peak-to-peak amplitudes of 0.23 and 0.26 µm with 4 µm pitch. A summary of the devices is presented in Table 1. Devices were heated into the isotropic phase of the FLC at 120 °C to remove any unwanted alignment memory in a hot stage (T95-PE, Linkam Scientific Instruments Ltd.) and mounted onto a polarising microscope (DM2700P, Leica Camera AG). Samples were controllably cooled into the nematic phase (*T*_NI_ = 100.8 °C) and SmA (*T*_SN_ = 86.3 °C) at 2 °C/min across phase transitions. For the phase transition into the SmC(*), the temperature of the device was cooled at 0.1 °C/min from 65 °C. Photographs of the LC phases were taken with a DSLR camera (D7100, Nikon). Response times and transmissions were measured with a photodiode with a green optical filter (made in-house) on a second microscope setup (Leica DM2700P microscope, FP90/FP82HT Mettler hot stage), with a programmable signal generator (WFG 500, FLC electronics).

## Supplementary information

Supplementary Information

## Data Availability

The datasets generated during and/or analysed during the current study are available from 10.5518/935.
