# Supplementary material for: A self-healing ferroelectric liquid crystal electro-optic shutter based on vertical surface-relief grating alignment
Source: Nat Commun. 2021 Aug 5;12:4717. doi: 10.1038/s41467-021-24953-5 (PMC8342423; doi:10.1038/s41467-021-24953-5)
Supplement: Supplementary file 1 — Supplementary Information [file 41467_2021_24953_MOESM1_ESM.pdf]

Supplementary Information for:
A self-healing ferroelectric liquid crystal electro-optic shutter based on vertical surface-relief grating alignment

Peter J. M. Wyatt[1], James Bailey[1,2], Mamatha Nagaraj[1] and J. Cliff Jones[1*]

Supplementary Notes

Additional information is presented regarding the switching mechanism of the Vertical Grating-Aligned Ferroelectric Liquid Crystal (VGA-FLC) device, indicating how the optimum response times are obtained for Fig. 8 in the main text. The aim is to induce a 90° twist of the **c**-director through the device with an applied electric field to switch to the ON state, and then the OFF state is re-obtained through either an applied restorative field or allowed to return with no additional pulse, the former being a faster optical response. The following assumptions are made on the mechanism. First, the total twist through the VGA-FLC increases with increasing field, where a twist of ±90° gives the maximum transmission of the same intensity. Second, further increasing the field beyond this point acts to unwind the induced twist, thus reducing the measured transmission, demonstrated by Fig. 2 in the main text. Also, the symmetry of the SmC* is such that **c**≠-**c** in the system. This results in either a right- or left-handed twist dependent on the sign of the electric field, demonstrated by Fig. 5 in the main text. This suggests that the OFF response cannot simply be obtained through reversing the sign of the field, as the **c**-director will switch from a +90° to -90° twist through an intermediate unwound state of 0°, for example, which are both ON states with an intermediate OFF state. Supplementary Fig. 1 demonstrates this, by showing the transmission response as a function of frequency. When the frequency is above 1kHz, the **c**-director twists in neither direction and so remains at 0° twist. The transmission data for the ON pulse is presented as a function of time in Supplementary Fig. 2, which shows how the **c**-director responds on the application of the field. Finally, the OFF responses are shown in Supplementary Fig.3, where the time of the pulse must be sufficient to restore the **c**-director to 0° twist, while not over-twisting in the opposite direction. Curves are shown for no restorative pulse (where the twist is allowed to unwind to relax the elastic deformation only), and for a pulse that is too short and too long. When the pulse is too short, the twist is not fully unwound, and so then unwinds at first quickly, and then slowly at the rate of the 0ms OFF pulse. The longer pulse achieves the minimum transmission quicker than the other two, however then increases in transmission again as the director twists back in the opposite direction. Supplementary Fig. 3b highlights how to obtain the fastest OFF times for a particular ON response, whereby the 0° twist state is achieved, without either under or over twist.

[1]School of Physics and Astronomy, University of Leeds, Leeds, UK. [2]Dynamic Vision Systems Ltd., Leeds Innovation Centre, Leeds, UK.
***e-mail: j.c.jones@leeds.ac.uk.**

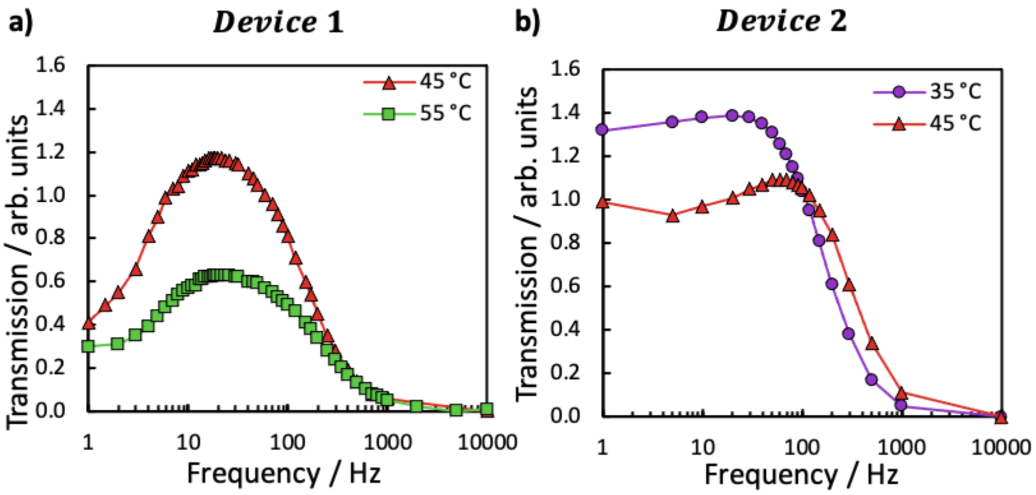

Supplementary Fig. 1. Frequency response of the vertical grating-aligned ferroelectric liquid crystal device (VGA-FLC). The frequency response of both Devices 1 & 2 displays a peak in transmission intensity and have a reduced or zero transmission when the frequency is above 1kHz, where the **c**-director remains untwisted and so is in the OFF state. Transmissions were measured with a photodiode with a green filter, and represent the average transmission over 256 averages, and so represent the average transmission over the ON and OFF states. The experimental uncertainties were calculated from the precision of the photodiode and signal noise, and are the size of or smaller than the data points. The trend lines are a guide for the eye. **a**, Device 1: T=55°C, V_{rms}=30V, and T=35°C, V_{rms}=35V. **b**, Device 2: V_{rms}=20V. The voltages are chosen as to provide the maximum transmission by twisting the **c**-director by 90°.

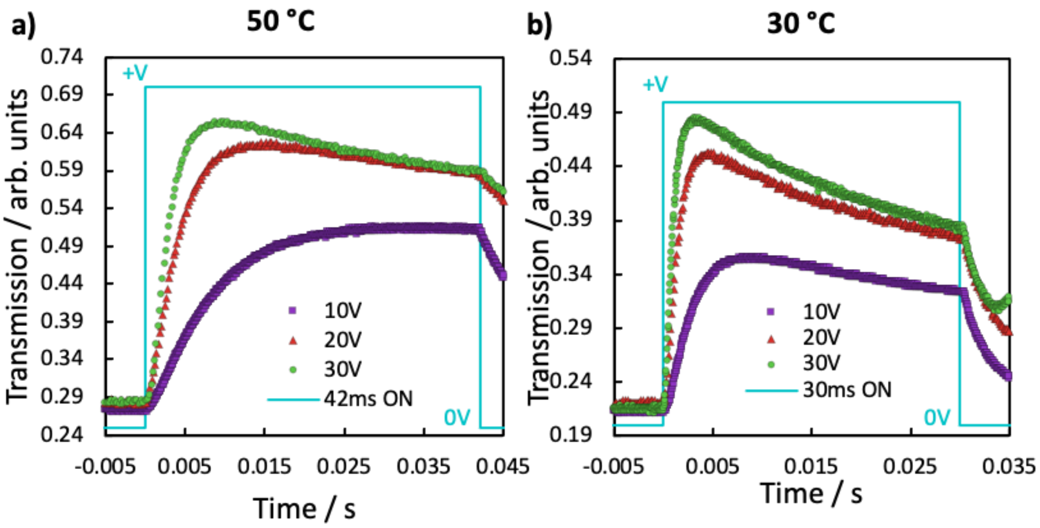

Supplementary Fig. 2. ON response of the c-director twist profile with applied DC voltage over time for Device 1. a, 50°C, 42ms DC pulse, and **b**, 30°C, 30ms DC pulse. The experimental uncertainties were calculated from the precision of the photodiode and signal noise, and are the size of, or smaller than, the data points.

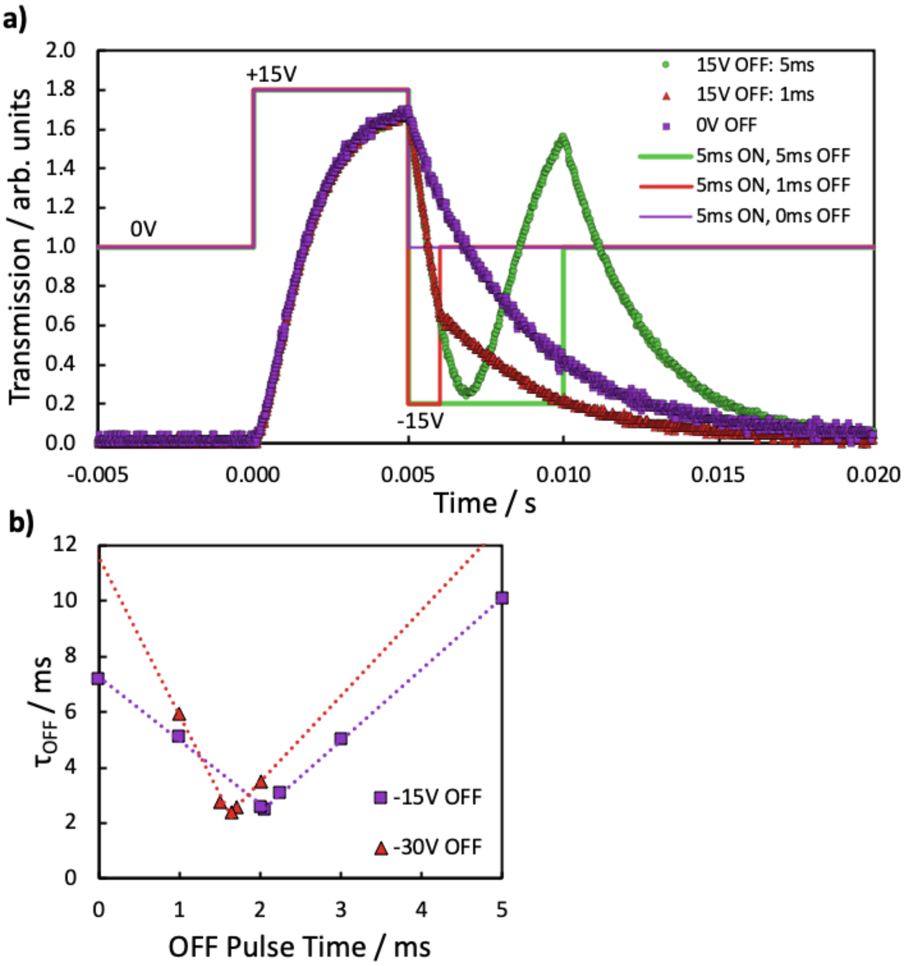

Supplementary Fig. 3. The OFF response for various pulse widths in VGA-FLC Device 1 at 50°C. a, A 15V ON pulse is applied for 5ms, and three OFF pulses are applied at -15V for 0ms, 1ms and 5ms, each represented by the coloured lines. **b**, The fastest OFF times obtained for a set of pulses and voltages. Off times at -15V measured after an ON pulse at +15V for 5ms, and those at -30V measured after a +30V pulse for 3ms. The dotted lines are guide to the eyes, and the experiments uncertainties were calculated from the precision of the photodiode and signal noise, and are approximately the same, or smaller than, the sizes of the data points.
